# VISTA^+^ follicular regulatory T cells modulate the function of effector immune cells: implications for ovarian cancer immune escape

**DOI:** 10.3389/fimmu.2025.1704048

**Published:** 2025-11-28

**Authors:** Yan Ma, Yidan Xu, Chunxia Yu, Huatuo Wu, Li Li

**Affiliations:** 1 The Third Clinical Medical College of Xinjiang Medical University (Affiliated Tumor Hospital), Urumqi, Xinjiang Uygur Autonomous Region, China; 2 Department of Gynecology, Affiliated Tumor Hospital of the Third Clinical Medical College, Xinjiang Medical University, Urumqi, Xinjiang Uygur Autonomous Region, China

**Keywords:** VISTA^+^ follicular regulatory T cells, ovarian cancer, CD8^+^ T cells, T helper cells, B cells, immune escape

## Abstract

**Background:**

As key regulatory cells, the impact of follicular regulatory T (Tfr) cells, whose function is regulated by V-domain Ig suppressor of T cell activation (VISTA), on downstream immune cells remains unclear. These cellular-level regulatory mechanisms are closely associated with the development of immune escape in ovarian cancer, a disease characterized by severe effector immune cell dysfunction in the tumor microenvironment. This study aims to elucidate the regulatory effects of VISTA^+^ Tfr cells on the functions of CD8^+^ T cells, CD4^+^CD25^−^ T cells, and B cells, and to reveal their significance in the immune escape of ovarian cancer.

**Methods:**

VISTA-overexpressing and silenced Tfr cell models were constructed *in vitro*. Through co-culture experiments, CFSE proliferation assays, ELISA, and flow cytometry were employed to investigate the effects of VISTA^+^ Tfr cells on CD8^+^ T cell proliferation, effector cytokine secretion, and activation status; their regulation of CD4^+^CD25^−^ T cell proliferation, cytokine secretion, and Th cell differentiation; and their impact on B cell proliferation and antibody secretion.

**Results:**

VISTA^+^ Tfr cells significantly inhibited CD8^+^ T cell proliferation, secretion of effector cytokines (IL-2, IFN-γ), and expression of the activation marker CD69, while upregulating their exhaustion molecules (PD-1, CTLA-4). They skewed the differentiation of CD4^+^CD25^−^ T cells toward a Th2 phenotype and suppressed Th1 and Th17 cell differentiation. Furthermore, they specifically inhibited IgE secretion by B cells, with no significant effect on other antibodies.

**Conclusion:**

VISTA^+^ Tfr cells multi-dimensionally suppress the function of effector immune cells, providing experimental evidence for the potential role of VISTA-targeted strategies in improving anti-tumor immune responses in ovarian cancer.

## 1 Introduction

Functional impairment of effector immune cells (such as cytotoxic T cells, helper T cells, and B cells) is a core driver of tumor immune escape, including that in ovarian cancer. Elucidating the cellular mechanisms underlying effector immune cell dysfunction is critical for understanding ovarian cancer immune evasion (1–3). Aberrant expression of immune checkpoint molecules and infiltration of immunosuppressive cells are two core drivers of tumor immune escape (4). V-domain Ig suppressor of T cell activation (VISTA), a novel immune checkpoint in the B7 family, exhibits significant mechanistic differences from programmed death 1 (PD-1)/cytotoxic T-lymphocyte-associated protein 4 (CTLA-4) (5, 6). VISTA not only suppresses activation signals by directly binding to receptors on T cells but also remodels the local immune microenvironment by regulating the function of antigen-presenting cells (7, 8). Previous studies have confirmed that VISTA is highly expressed in solid tumors such as lung cancer and renal cell carcinoma, and is closely associated with tumor stage and patient prognosis (9, 10). However, its specific role in ovarian cancer, particularly its regulatory effects on immune cell subsets, remains poorly understood.

Follicular regulatory T (Tfr) cells, serving as key regulators bridging cellular and humoral immunity, uniquely co-express FOXP3 (a core transcription factor for immunosuppressive function) and CXCR5 (a lymph follicle homing receptor). They play distinct roles in secondary lymphoid organs and the tumor microenvironment (11–13). Under physiological conditions, Tfr cells maintain the balance of B cell antibody secretion by suppressing the overactivation of follicular helper T cells. In the tumor microenvironment, increased Tfr cell infiltration is known to enhance immunosuppression via IL-10 and TGF-β secretion. However, the molecular mechanisms regulating Tfr cell function in cellular models (relevant to ovarian cancer) remain unclear (14–16). Notably, Tfr cells highly express various immune checkpoint molecules on their surface, suggesting they may serve as crucial “effector carriers” of immune checkpoint signals. However, whether VISTA, as a novel checkpoint, participates in ovarian cancer immune escape by regulating Tfr cell function remains an unresolved question (17, 18).

The functional integrity of effector immune cells is central to anti-tumor immune responses: The cytotoxic activity of CD8^+^ T cells serves as the primary force for eliminating tumor cells, and their functional exhaustion (manifested by restricted proliferation, reduced secretion of effector factors, and upregulation of exhaustion molecules such as PD-1) directly impairs immune responses (19, 20). Imbalance in the Th1/Th2/Th17 subsets differentiated from CD4^+^CD25^−^ T cells (such as a Th2 bias) disrupt the immunoregulatory network and promotes tumor progression (21–23). B cells participate in humoral immunity through antibody secretion (e.g., IgE-mediated antibody-dependent cell-mediated cytotoxicity), and their functional abnormalities are linked to tumor microenvironment remodeling in ovarian cancerningonm this link has not been tested in cellular models of VISTA^+^ Tfr cell regulation. However, current research has not yet clarified whether VISTA^+^ Tfr cells promote immune escape in ovarian cancer in multiple dimensions by targeting these effector cells (24, 25). Investigating whether VISTA^+^ Tfr cells selectively suppress the cytotoxicity of CD8^+^ T cells, regulate the differentiation bias of CD4^+^CD25^−^ T cells, and influence the antibody secretion profile of B cells will provide new perspectives for understanding the complexity of the immune microenvironment in ovarian cancer (26–28).

However, current research has not yet clarified whether VISTA^+^ Tfr cells regulate these effector cells at the cellular level to support the immune escape of ovarian cancer. This study focuses on *in vitro* cell interactions and provides a cellular-level basis for understanding the complexity of the immune microenvironment in ovarian cancer. This study focuses on the regulatory effects of VISTA^+^ Tfr cells on CD8^+^ T cells, CD4^+^CD25^−^ T cells, and B cells. Utilizing *in vitro* co-culture models combined with functional assays, we systematically analyze the impact of VISTA^+^ Tfr cells on effector cell proliferation, cytokine secretion, activation phenotypes, and differentiation fates—aiming to elucidate the cellular mechanisms that may underlie VISTA^+^ Tfr cell-mediated immune escape in ovarian cancer. The findings are expected not only to reveal a new paradigm of synergistic action between immune checkpoints and inhibitory T cell subsets but also to provide experimental evidence and theoretical support for developing VISTA-targeted combination immunotherapy strategies, such as combined PD-1 inhibition or Tfr cell-targeted interventions.

## 2 Methods

### 2.1 Cell culture

Peripheral blood mononuclear cells (PBMCs) were isolated from healthy human donors using human peripheral blood lymphocyte separation solution (Solarbio, P8610). Fresh anticoagulated whole blood from healthy donors was diluted with an equal volume of RPMI 1640 medium (GIBCO, 11875101). Then, 6 mL of the diluted blood sample was carefully layered onto 3 mL of separation solution in a 15 mL centrifuge tube (Excell Bio, CS015-0001). The tube was centrifuged at 2000 rpm (using a benchtop low-speed centrifuge, Shanghai Feige, DK-80) for 20 min at room temperature. The intermediate white buffy coat layer containing PBMCs was collected, washed twice with 5 mL phosphate-buffered saline (PBS, centrifuged at 1500 rpm for 5 min each time), and finally resuspended in RPMI 1640 medium for subsequent use.

#### 2.1.1 Tfr cell

Naive CD4^+^ T cells were isolated using the Naive CD4^+^ T Cell Isolation Kit II (Miltenyi, 130-094-131). 10^7^ PBMCs were resuspended in 40 μL of separation buffer, followed by sequential addition of 10 μL of biotin-antibody cocktail and 20 μL of microbeads, with each incubation step performed for 5 min at room temperature. LS Columns (Miltenyi, 130-042-401) were placed on the OctoMACS™ Separator (Miltenyi, 130-042-108), pre-rinsed with 3 mL of separation buffer, and then loaded with the cell suspension. Unlabeled cells were collected, and the column was rinsed with an additional 3 mL of buffer. Finally, target cells were eluted using 1 mL of Bead Release Reagent buffer, washed with PBS, and prepared for subsequent use. Naive CD4^+^ T cells were induced to differentiate into FOXP3^+^CXCR5^+^ T lymphocytes by culturing for 5 days in RPMI 1640 medium supplemented with fetal bovine serum (FBS, Excell Bio, FND500), penicillin-streptomycin (GIBCO, 15070-063), CD3 monoclonal antibody (MultiSciences, F1100300, 0.5 μg/mL), CD28 antibody (Santa Cruz, sc-53876, 0.5 μg/mL), recombinant interleukin-2 (IL-2) protein (proteintech, HZ-1015, 5 ng/mL), recombinant TGF-β1 protein (proteintech, HZ-1011, 5 ng/mL), and recombinant IL-23 protein (proteintech, HZ-1254, 25 ng/mL). For flow cytometric sorting of Tfr cells, the induced cells were collected and adjusted to a concentration of 1×10^7^ cells/mL in serum-free medium. They were then stained with CD25 antibody (BD, 557753), CD127 antibody (BD, 557938), CXCR5 antibody (Thermo Fisher, 17-9185-42), and VISTA antibody (Thermo Fisher, 25-1088-42), followed by incubation for 30 min at 4 °C in the dark. CD25^+^CD127^−^CXCR5^+^VISTA^+^ and CD25^+^CD127^−^CXCR5^+^VISTA^−^ cells were sorted using a flow cytometer (BD, FACSVerse).

#### 2.1.2 CD8^+^T cell

For every 10^8^ PBMCs, add 40 μL of separation buffer to resuspend the cells. Add 10 μL of REAlease CD8-Biotin and mix thoroughly, then incubate at room temperature for 5 min. Next, add 20 µL of CD8 Anti-Biotin MicroBeads, mix well, and incubate at room temperature for another 5 min. No washing step is required. Place the LS Column on the OctoMACS. Separator magnet. Pre-rinse the column with 3 mL of separation buffer. Apply 3 mL of the above cell suspension to the column, and place a collection tube at the bottom to collect the unlabeled cells. Then, rinse the column with an additional 3 mL of separation buffer. Remove the column from the magnet and place it over a collection tube. Add 1 mL of Bead Release Reagent buffer, and immediately push the plunger to elute the target cells. Centrifuge the eluted cells at 1500 rpm for 5 min, wash with PBS, and reserve for subsequent use.

#### 2.1.3 Helper T lymphocytes

The magnetically sorted CD4^+^ T lymphocytes were resuspended in PBS at a concentration of 1×10^7^ cells/mL. CD25 antibody was added, followed by incubation at 4 °C in the dark for 30 min. CD25^−^CD4^+^ T lymphocytes were then sorted using a flow cytometer. The sorted cells were centrifuged at 1500 rpm for 5 min, the supernatant was discarded, and the cell pellet was resuspended in RPMI 1640 medium supplemented with 10% FBS for subsequent use.

#### 2.1.4 B lymphocytes

For every 10^8^ PBMCs, add 40 μL of separation buffer to resuspend the cells. Add 10 μL of REAlease CD19-Biotin and mix well, then incubate at room temperature for 5 min. Next, add 20 µL of CD19 Anti-Biotin MicroBeads, mix thoroughly, and incubate at room temperature for another 5 min. Place an LS Column on the OctoMACSu Separator magnet. Pre-rinse the column with 3 mL of separation buffer, then apply the cell suspension. Collect the unlabeled cells, and rinse the column with an additional 3 mL of buffer. Remove the column from the magnet, add 1 mL of Bead Release Reagent, and immediately push the plunger to elute the B lymphocytes. Centrifuge the eluted cells at 1500 rpm for 5 min, wash with PBS, and reserve for subsequent use.

### 2.2 Construction of VISTA-overexpressing/silenced Tfr cells

VISTA-overexpressing and VISTA-silencing lentiviruses were both purchased from GeneChem (pLVX-VISTA-IRES-ZsGreen1 for overexpression and pLVX-shVISTA-IRES-Puro for silencing). The experiment was divided into three groups: Group M (conventional medium), Group A (conventional medium + HiTransG A), and Group P (conventional medium + HiTransG P), to evaluate the effects of conventional culture and two types of infection enhancers on viral infection efficiency. For viral transfection, a cell suspension at 5×10^4^ cells/mL was prepared in complete medium, and 100 μL was added per well to a 96-well plate (including 3 control wells). The negative control virus was thawed on ice and diluted with serum-free medium to concentrations of 2×10^7^, 1×10^7^, and 1×10^6^ TU/mL (50 μL each). The supernatant in the wells was aspirated, and corresponding medium, virus, and infection enhancer were added (MOI = 20, Polybrene, final concentration 8 μg/mL), The mixture was thoroughly mixed and cultured. After 12 h, the medium was replaced with normal medium. At 72 h, when fluorescence intensity was high, microscopic examination was performed (The markers used are ZsGreen1 for VISTA overexpression vector). Conditions and multiplicity of infection yielding approximately 80% infection efficiency and good cell growth were selected for subsequent experiments (**Supplementary Figure S1**).

### 2.3 Cell proliferation assay

Induced Tfr cells were sorted using flow cytometry to obtain VISTA^+^ Tfr cells and VISTA^−^ Tfr cells. For VISTA^−^ Tfr cells, the following groups were established: blank control group, OE negative control group (transfected with negative control virus), and VISTA overexpression group (transfected with LV-VISTA overexpression lentivirus). For VISTA^+^ Tfr cells, the following groups were established: blank control group, shRNA negative control group (transfected with negative control virus), and VISTA shRNA intervention group (transfected with VISTA-RNAi). Prior to viral transfection, cells from each group were stained using the CellTrace™ CFSE Cell Proliferation Kit (Thermo Fisher, C34570) at a concentration of 0.5 μM per 1×10^6^ cells/mL. After lentiviral infection, cells from each group were either cultured alone, co-cultured with CD8^+^ T lymphocytes (CD8^+^ T: Tfr = 4:1), co-cultured with CD25^−^CD4^+^ T lymphocytes (CD25^−^CD4^+^ T: Tfr = 4:1), or co-cultured with B lymphocytes (B: Tfr = 4:1; with stimulation using 2 μg/mL anti-CD3 (MultiSciences, F1100300) and 5 μg/mL anti-IgM during co-culture) for 5 days. After the culture period, cells from each group were collected, washed once with PBS, resuspended in 500 μL PBS, and analyzed for CFSE fluorescence intensity by flow cytometry.

### 2.4 Enzyme-Linked Immunosorbent Assay

VISTA^+^ Tfr cells and VISTA^−^ Tfr cells, sorted by flow cytometry after induction, were divided into blank control, negative control, and target intervention groups. After lentiviral infection, cells from each group were cultured alone or co-cultured with CD8^+^ T lymphocytes, CD25^−^CD4^+^ T lymphocytes, or B lymphocytes, respectively. After the culture period, supernatants from each group were collected and analyzed by ELISA using a microplate reader (Bio-Rad, xMark™). For co-culture groups with CD8^+^ T lymphocytes, levels of IL-2, Interferon-γ (IFN-γ), granzyme B, and perforin were detected (MultiSciences, EK102, EK180, EK158; CUSABIO, CSB-E09313h). For co-culture groups with CD25^−^CD4^+^ T lymphocytes, levels of IL-1α, IL-2, and tumor necrosis factor-α(TNF-α) were measured (MultiSciences, EK101A, EK102, EK182). For co-culture groups with B lymphocytes, levels of IgA, IgE, IgM, and IgG were quantified (MultiSciences, EK174-48, EK175-48, EK176-96, EK171-96). All procedures were performed according to the manufacturers’ instructions.

### 2.5 Flow cytometry

After co-culture of Tfr cells with CD8^+^ T cells, cells from each group were collected and resuspended in PBS at a concentration of 1×10^7^ cells/mL. 100 μL of cell suspension (containing 1×10^6^ cells) was transferred to a flow cytometry tube, followed by sequential addition of 5 μL each of CD8-PerCP-Cy™5.5 (BD, 565310), CD69-PE/Cyanine7 (Elabscience, E-AB-F1138H), lymphocyte-activation gene 3 (LAG3)-FITC (MultiSciences, F1122301), T-cell immunoglobulin and mucin-domain containing-3 (TIM-3)-PE (MultiSciences, F2136602), PD-1-APC-Cy7 (MultiSciences, F1127906), and CTLA-4-APC (MultiSciences, F1115203) antibodies. The mixture was vortexed and incubated at room temperature in the dark for 30 min. Cells were washed twice with PBS, resuspended in 500 μL PBS, filtered through a 200-mesh sieve, and analyzed by flow cytometry within 2 h to detect the expression of CD8^+^, CD8^+^CD69^+^, CD8^+^LAG3^+^, CD8^+^TIM-3^+^, CD8^+^PD-1^+^, and CD8^+^CTLA-4^+^ cells. After co-culture of Tfr cells with CD25^−^CD4^+^ T cells, cells from each group were collected and resuspended in PBS at a concentration of 1×10^7^ cells/mL. 100 μL of cell suspension (containing 1×10^6^ cells) was transferred to a flow cytometry tube and stimulated with phorbol 12-myristate 13-acetate (50 ng/mL), ionomycin (1 μg/mL), and monensin (1.7 μg/mL, Elabscience, E-CK-A013) for 4 h. Cells were collected, centrifuged at 1500 rpm for 5 min, and the supernatant was discarded. The cell pellet was resuspended in 100 μL PBS, and 5 μL of CD4-PerCP-Cy™5.5 antibody was added, followed by incubation at 4°C in the dark for 30 min. After washing, cells were fixed with 500 μL Fixation Buffer (Elabscience, E-CK-A109) at 4°C in the dark for 20 min, centrifuged at 1500 rpm for 5 min, and the supernatant was discarded. Then, 500 μL Permeabilization Buffer (Elabscience, E-CK-A109) was added for permeabilization at 4°C in the dark for 15 min. Cells were centrifuged at 1500 rpm for 5 min, the supernatant was discarded, and the pellet was resuspended in 100 μL Permeabilization Buffer. Then, 5 μL each of IL-4-APC (MultiSciences, F11IL403), IFN-γ-PE (MultiSciences, F11IFNG02), and IL-17A-FITC (Elabscience, E-AB-F1173C) antibodies were added, followed by incubation at 4°C in the dark for 30 min. Cells were washed twice with PBS, resuspended in 500 μL PBS, filtered through a 200-mesh sieve, and analyzed by flow cytometry within 2 h to determine the proportions of Th1 (CD4^+^IFN-γ^+^), Th2 (CD4^+^IL-4^+^), and Th17 (CD4^+^IL-17^+^) cells.

### 2.6 Statistical analysis

Statistical analyses were performed using SPSS 19.0. Normally distributed measurement data are presented as mean ± standard deviation ( $\bar{x}$ ± s). All experiments were performed with biological replicates (n=3) (no technical repeats were used, as each replicate represented an independent donor or cell isolation). One-way analysis of variance (ANOVA) was used for comparisons among multiple groups, followed by least significant difference test (for homogeneous variance) or Dunnett’s T3 test (for heterogeneous variance) for *post-hoc* pairwise comparisons. Non-normally distributed measurement data are expressed as median (interquartile range) [M (P25-P75)]. The Kruskal-Wallis H test was employed for comparisons among multiple groups, followed by the Kruskal-Wallis one-way ANOVA (k-sample) for multiple comparisons. A P-value < 0.05 was considered statistically significant. Graphs were generated using GraphPad Prism 5.0.

## 3 Results

### 3.1 VISTA enhances the inhibitory effect of Tfr cells on CD8^+^ T cell proliferation and effector cytokine secretion

**Figure 1:**
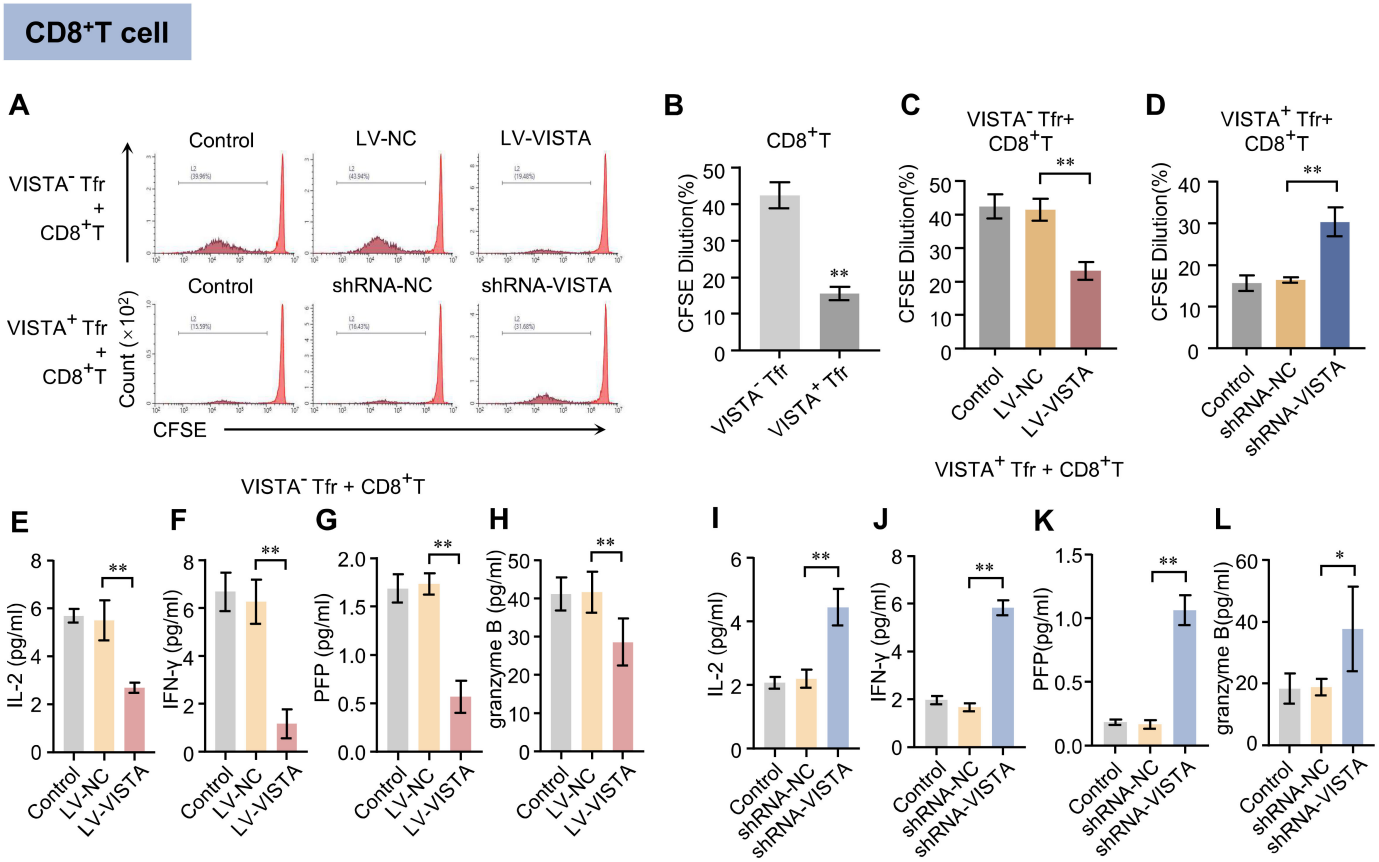
Effects of VISTA^+^/VISTA^−^ Tfr cells on CD8^+^ T cell proliferation and cytokine secretion. After co-culture of VISTA^+^ or VISTA^−^ Tfr cells with CD8^+^ T cells (CD8^+^ T cells: Tfr cells = 4:1): **(A)** Representative flow cytometry histogram of CFSE proliferation assay for co-cultured cells. The x-axis represents CFSE fluorescence intensity, and the y-axis represents cell count (n=3). **(B)** Quantitative analysis of CFSE fluorescence intensity in VISTA^−^ Tfr + CD8^+^ T cell group versus VISTA^+^ Tfr + CD8^+^ T cell group. **(C)** Quantitative analysis of CFSE fluorescence intensity after co-culture of CD8^+^ T cells with VISTA^−^ Tfr cells overexpressing VISTA (LV-VISTA). **(D)** Quantitative analysis of CFSE fluorescence intensity after co-culture of CD8^+^ T cells with VISTA^+^ Tfr cells with VISTA silenced (VISTA-shRNA). **(E–L)** Levels of IL-2, IFN-γ, perforin (PFP), and granzyme B in culture supernatants of VISTA^−^ Tfr + CD8^+^ T cell group **(E–H)** and VISTA^+^ Tfr + CD8^+^ T cell group **(I–L)** as detected by ELISA (n=3). The statistical results are expressed as the mean ± SD, *p < 0.05, **p < 0.01.

To determine the effects of VISTA^+^ and VISTA^−^ Tfr cells on CD8^+^ T cell proliferation, flow-sorted cells were co-cultured with CD8^+^ T cells, respectively. When co-cultured with VISTA^+^ Tfr cells, CD8^+^ T cells showed a significantly lower proportion of 5,6-Carboxyfluorescein diacetate succinimidyl ester (CFSE) fluorescence decay compared to the VISTA^−^ Tfr cell co-culture group (**Figures 1A, B**). Functional validation demonstrated that after VISTA overexpression in VISTA^−^ Tfr cells, the proportion of CFSE fluorescence decay in CD8^+^ T cells was significantly reduced compared to the blank and negative control groups (**Figure 1C**). Following VISTA silencing in VISTA^+^ Tfr cells, this proportion was significantly increased compared to the blank and negative control groups (**Figure 1D**). These results indicate that VISTA enhances the inhibitory effect of Tfr cells on CD8^+^ T lymphocyte proliferation. ELISA assays revealed that CD8^+^ T cells in the VISTA^+^ Tfr cell co-culture group had a significantly reduced capacity to secrete the aforementioned cytokines compared to the VISTA^−^ Tfr cell co-culture group (**Supplementary Figures 2A-D**). Further functional validation showed that after VISTA overexpression in VISTA^−^ Tfr cells, the levels of IL-2, IFN-γ, perforin, and granzyme B secreted by CD8^+^ T cells were significantly decreased compared to the control group (**Figures 1E–H**). In contrast, after VISTA silencing in VISTA^+^ Tfr cells, the levels of cytokines secreted by CD8^+^ T cells were significantly increased compared to the control group (**Figures 1I–L**). These results demonstrate that VISTA enhances the inhibitory effect of Tfr cells on effector cytokine secretion by CD8^+^ T cells, which is consistent with previous findings that VISTA blockade promotes effector cytokine secretion (29).

### 3.2 VISTA regulates the activation phenotype and immune checkpoint molecule expression of CD8^+^ T cells through Tfr cells

**Figure 2:**
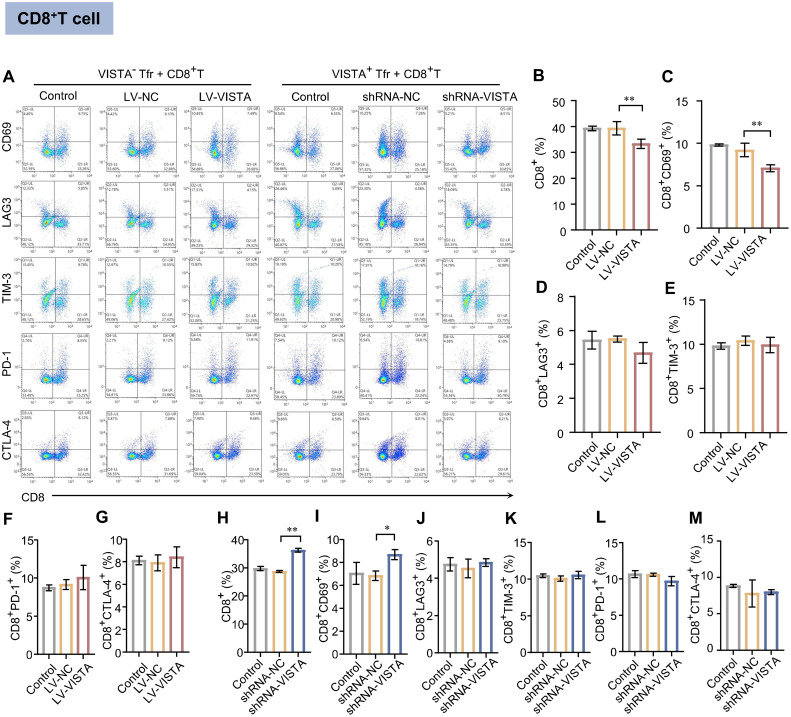
Effects of VISTA^+^/VISTA^−^ Tfr cells on the expression of activation markers and exhaustion-associated immune checkpoints on CD8^+^ T cells. **(A)** Flow cytometry scatter plots showing the effects of VISTA^+^/VISTA^−^ Tfr cells in the co-culture system on the expression of the activation marker CD69 and exhaustion-associated immune checkpoints (LAG3, TIM-3, PD-1, CTLA-4) on CD8^+^ T cells (n=3). **(B–G)** Quantitative bar graphs showing the expression proportions of surface markers on CD8^+^ T cells after co-culture with VISTA^−^ Tfr cells or VISTA^−^ Tfr cells overexpressing VISTA (LV-VISTA). **(H–M)** Quantitative bar graphs showing the expression proportions of surface markers on CD8^+^ T cells after co-culture with VISTA^+^ Tfr cells or VISTA^+^ Tfr cells with VISTA silenced (shRNA-VISTA). The statistical results are expressed as the mean ± SD, *p < 0.05, **p < 0.01.

Using an established co-culture system, we examined the effects of VISTA^+^/VISTA^−^ Tfr cells on the expression of the activation marker CD69 and exhaustion-associated immune checkpoint molecules (LAG3, TIM-3, PD-1, CTLA-4) on CD8^+^ T cells (**Figure 2A**) (26, 27, 30). Flow cytometric analysis revealed that compared to the VISTA^−^ Tfr cell co-culture group, CD8^+^ T cells co-cultured with VISTA^+^ Tfr cells showed significantly downregulated expression of the early activation marker CD69 (reduced proportion of CD8^+^CD69^+^ cells), while the proportions of PD-1^+^ and CTLA-4^+^ cells were increased. No statistically significant differences were observed in the expression of LAG3 and TIM-3 (**Supplementary Figures 2E-J**). Further functional validation demonstrated that after VISTA overexpression in VISTA^−^ Tfr cells, the proportion of CD8^+^CD69^+^ cells decreased compared to the blank and negative control groups, while the proportions of PD-1^+^ and CTLA-4^+^ cells increased (**Figures 2B–G**). Conversely, following VISTA silencing in VISTA^+^ Tfr cells, the proportion of CD8^+^CD69^+^ cells increased compared to the control group, while the proportion of PD-1^+^ cells decreased. No significant changes were observed in the expression of CTLA-4, LAG3, or TIM-3 (**Figures 2H–M**). These results indicate that VISTA, via Tfr cells, suppresses the early activation of CD8^+^ T cells and specifically upregulates the expression of PD-1 and CTLA-4, which is consistent with previous research demonstrating that immune checkpoint molecules collaboratively contribute to T cell exhaustion (31).

### 3.3 VISTA enhances the inhibitory effect of Tfr cells on the proliferation and pro-inflammatory cytokine secretion of CD4^+^CD25^−^ T cells

**Figure 3:**
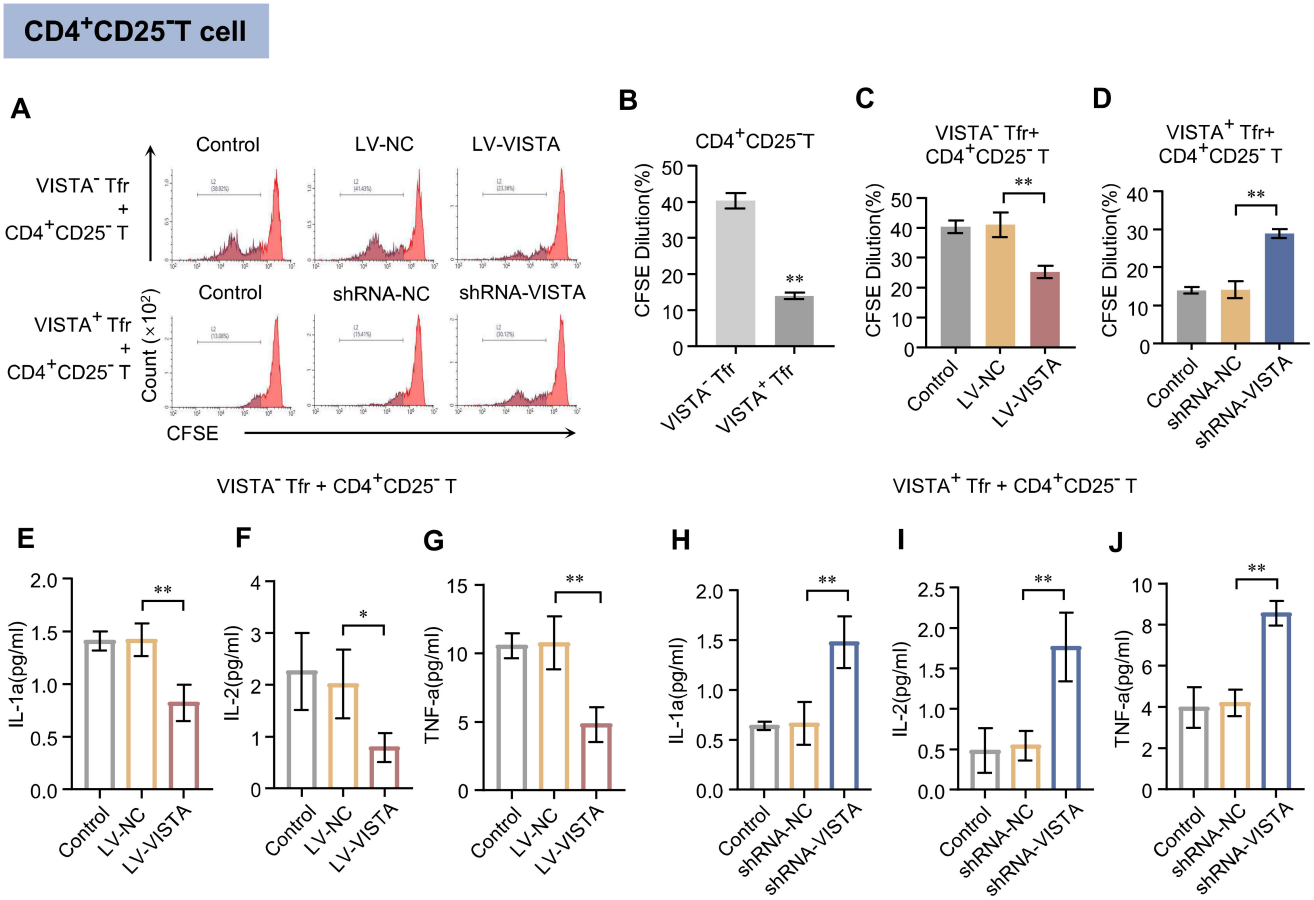
Effects of VISTA^+^/VISTA^−^ Tfr cells on CD4^+^CD25^−^ T cell proliferation and cytokine secretion. After co-culture of VISTA^+^ or VISTA^−^ Tfr cells with CD4^+^CD25^−^ T lymphocytes (CD25^−^CD4^+^ T: Tfr = 4:1): **(A)** Representative flow cytometry histogram of CFSE proliferation assay for co-cultured cells. The x-axis represents CFSE fluorescence intensity, and the y-axis represents cell count (n=3). **(B)** Quantitative analysis of CFSE fluorescence intensity in the VISTA^−^ Tfr + CD25^−^CD4^+^ T cell group versus the VISTA^+^ Tfr + CD25^−^CD4^+^ T cell group. **(C)** Quantitative analysis of CFSE fluorescence intensity after co-culture of CD25^−^CD4^+^ T cells with VISTA^−^ Tfr cells overexpressing VISTA (LV-VISTA). **(D)** Quantitative analysis of CFSE fluorescence intensity after co-culture of CD25^−^CD4^+^ T cells with VISTA^+^ Tfr cells with VISTA silenced (VISTA-shRNA). **(E–J)** Levels of IL-1α, IL-2, and TNF-α in culture supernatants of the VISTA^−^ Tfr + CD25^−^CD4^+^ T cell group **(E–G)** and the VISTA^+^ Tfr + CD25^−^CD4^+^ T cell group **(H–J)** as detected by ELISA (n=3). The statistical results are expressed as the mean ± SD, *p < 0.05, **p < 0.01.

VISTA^+^ Tfr cells and VISTA^−^ Tfr cells were separately co-cultured with CD4^+^CD25^−^ T lymphocytes to assess their proliferation (**Figure 3A**). The results showed that CD4^+^CD25^−^ T lymphocytes in the VISTA^−^ Tfr cell co-culture group proliferated significantly faster than those in the VISTA^+^ Tfr cell co-culture group (**Figure 3B**). Functional validation demonstrated that after VISTA overexpression in Tfr cells, the inhibitory effect on CD4^+^CD25^−^ T lymphocyte proliferation was enhanced (**Figure 3C**). In contrast, following VISTA silencing, the inhibitory effect was attenuated (**Figure 3D**). These findings indicate that VISTA enhances the inhibitory effect of Tfr cells on CD4^+^CD25^−^ T lymphocyte proliferation. The supernatants from the co-culture systems were collected to measure the levels of IL-1α, IL-2, and TNF-α. The results revealed that the levels of these cytokines in the supernatant of CD4^+^CD25^−^ T lymphocytes co-cultured with VISTA^+^ Tfr cells were significantly lower than those in the VISTA^−^ Tfr co-culture group (**Supplementary Figures 3A-C**). After VISTA overexpression in VISTA^−^ Tfr cells, the levels of IL-1α, IL-2, and TNF-α in the co-culture supernatant were significantly reduced (**Figure 3E–G**). Following VISTA silencing in VISTA^+^ Tfr cells, the levels of these cytokines were significantly increased (**Figures 3H–J**). These results demonstrate that VISTA enhances the inhibitory effect of Tfr cells on the secretion of pro-inflammatory cytokines by CD4^+^CD25^−^ T lymphocytes.

### 3.4 VISTA regulates Tfr cells to direct CD4^+^CD25^−^ T cell differentiation toward Th2 while suppressing Th1/Th17 differentiation

**Figure 4:**
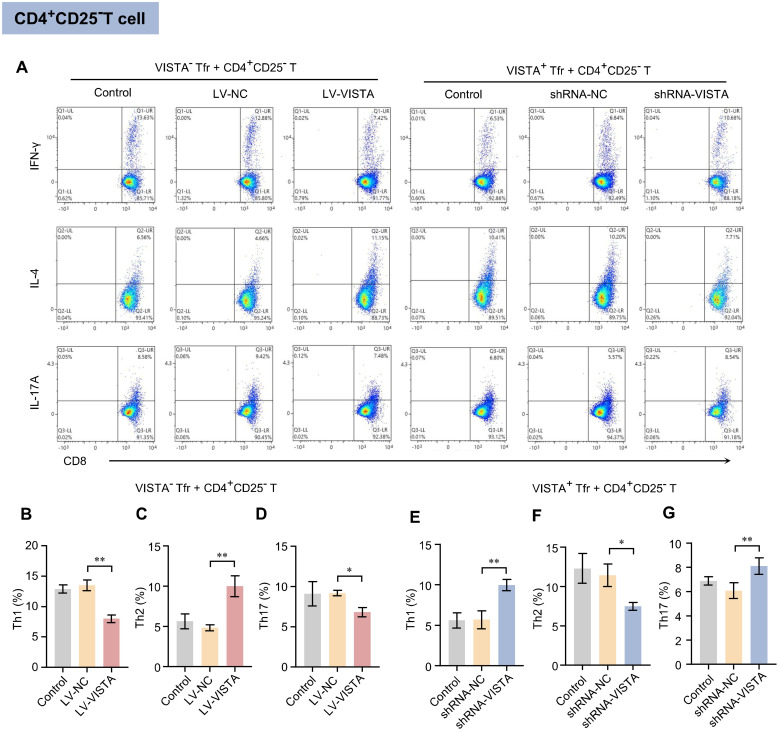
Effects of VISTA^+^/VISTA^−^ Tfr cells on CD25^−^CD4^+^ T cell differentiation. **(A)** Flow cytometry scatter plots showing the effects of VISTA^+^/VISTA^−^ Tfr cells in the co-culture system on the expression of differentiation markers IFN-γ, IL-4, and IL-17A on CD25^−^CD4^+^ T cells (n=3). **(B–D)** Quantitative bar graphs showing the expression proportions of Th1, Th2, and Th17 cells after co-culture of CD25^−^CD4^+^ T cells with VISTA^−^ Tfr cells or VISTA^−^ Tfr cells overexpressing VISTA (LV-VISTA). **(E–G)** Quantitative bar graphs showing the expression proportions of Th1, Th2, and Th17 cells after co-culture of CD25^−^CD4^+^ T cells with VISTA^+^ Tfr cells or VISTA^+^ Tfr cells with VISTA silenced (shRNA-VISTA). The statistical results are expressed as the mean ± SD, *p < 0.05, **p < 0.01, ns, no significant difference.

Flow cytometric analysis of CD4^+^CD25^−^ T lymphocyte differentiation phenotypes revealed that, compared to the VISTA^−^ Tfr cell co-culture group, the VISTA^+^ Tfr cell co-culture group showed a significantly lower proportion of Th1 cells (IFN-γ^+^) and a significantly higher proportion of Th2 cells (IL-4^+^), while the proportion of Th17 cells (IL-17^+^) showed no statistical difference (**Figure 4A**; **Supplementary Figures 3D-F**). Functional validation experiments further confirmed that after VISTA overexpression in VISTA^−^ Tfr cells, the proportions of Th1 and Th17 cells in the co-culture system decreased compared to the blank and negative control groups, while the proportion of Th2 cells increased (**Figures 4B–D**). Conversely, following VISTA silencing in VISTA^+^ Tfr cells, the proportions of Th1 and Th17 cells increased compared to the control group, while the proportion of Th2 cells decreased (**Figures 4E–G**). These results indicate that VISTA can specifically regulate the differentiation direction of CD4^+^CD25^−^ T cells through Tfr cells, significantly promoting their differentiation toward the immunosuppressive Th2 subset while inhibiting the differentiation of the anti-tumor Th1 and Th17 subsets. This suggests that this regulatory pattern may be an important mechanism underlying the imbalance in the ovarian cancer immune microenvironment.

### 3.5 VISTA expression in Tfr cells selectively inhibits IgE secretion by B cells

**Figure 5:**
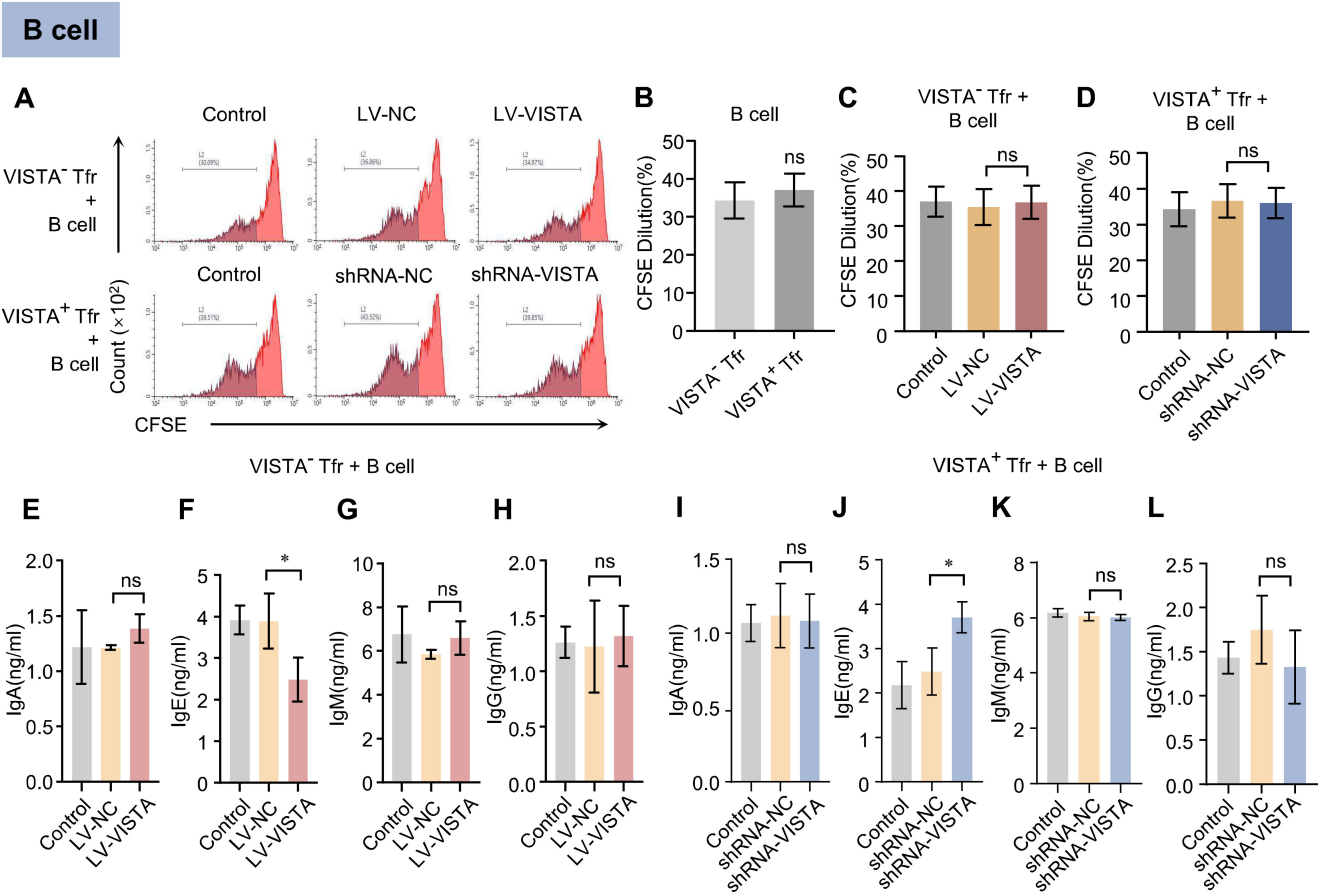
Regulatory effects of VISTA^+^/VISTA^−^ Tfr cells on B cell proliferation and antibody secretion. After co-culture of VISTA^+^ or VISTA^−^ Tfr cells with B lymphocytes (B: Tfr = 4:1): **(A)** Representative flow cytometry histogram of CFSE proliferation assay for co-cultured cells. The x-axis represents CFSE fluorescence intensity, and the y-axis represents cell count (n=3). **(B)** Quantitative analysis of CFSE fluorescence intensity in the VISTA^−^ Tfr + B cell group versus the VISTA^+^ Tfr + B cell group. **(C)** Quantitative analysis of CFSE fluorescence intensity after co-culture of B cells with VISTA^−^ Tfr cells overexpressing VISTA (LV-VISTA). **(D)** Quantitative analysis of CFSE fluorescence intensity after co-culture of B cells with VISTA^+^ Tfr cells with VISTA silenced (VISTA-shRNA). **(E–L)** Levels of IgA, IgE, IgM, and IgG in culture supernatants of the VISTA^−^ Tfr + B cell group **(E–H)** and the VISTA^+^ Tfr + B cell group **(I–L)** as detected by ELISA (n=3). The statistical results are expressed as the mean ± SD, *p < 0.05, **p < 0.01.

After co-culture of Tfr cells with B lymphocytes, B cell proliferation was assessed. The results showed no significant differences in the proportion of CFSE fluorescence decay among B cells co-cultured with VISTA^+^ Tfr cells, VISTA^−^ Tfr cells, or the corresponding VISTA-silenced and VISTA-overexpressing groups (**Figures 5A–D**). This indicates that VISTA expression levels in Tfr cells do not significantly regulate B lymphocyte proliferation. Further ELISA detection of antibody secretion levels in the co-culture supernatants revealed that IgE secretion by B cells co-cultured with VISTA^+^ Tfr cells was significantly lower than that in the VISTA^−^ Tfr cell co-culture group (**Supplementary Figures 4A-D**). After VISTA overexpression in VISTA^−^ Tfr cells, IgE secretion levels were significantly reduced compared to the negative control group (**Figure 5E**). Following VISTA silencing via shRNA in VISTA^+^ Tfr cells, IgE secretion levels were significantly increased compared to the negative control group (**Figure 5F**). In contrast, the secretion levels of IgA, IgM, and IgG showed no significant differences among the groups. These results demonstrate that VISTA specifically enhances the inhibitory effect of Tfr cells on IgE secretion by B lymphocytes, with no significant impact on the secretion of other antibody types.

## 4 Discussion

The cytotoxic function of CD8^+^ T cells and the helper immune effects of CD4^+^CD25^−^ T cells are central to the body’s anti-tumor immunity, and their functional impairment is a key feature of immune escape in ovarian cancer (32–34). This study found that VISTA^+^ Tfr cells significantly inhibit CD8^+^ T cell proliferation and effector factor secretion (IL-2, IFN-γ) *in vitro*, downregulate the expression of the activation marker CD69, and simultaneously upregulate exhaustion-related molecules such as PD-1 and CTLA-4. These results are consistent with previous studies demonstrating that VISTA suppresses T cell cytotoxicity (18, 29). However, this study is the first to confirm that Tfr cells are key mediators of VISTA’s inhibitory effect on CD8^+^ T cells, and that this inhibition can be reversed by silencing VISTA, suggesting that targeting VISTA may restore CD8^+^ T cell anti-tumor activity—with potential implications for ovarian cancer. For CD4^+^CD25^−^ T cells, VISTA^+^ Tfr cells not only inhibit their proliferation and the secretion of pro-inflammatory cytokines (IL-1α, IL-2, TNF-α) but also significantly skew their differentiation bias, promoting Th2 cell differentiation while suppressing Th1 and Th17 cells. Th1 cells primarily mediate cellular immunity through IFN-γ secretion, Th17 cells are involved in inflammatory anti-tumor responses, while Th2 cells tend to promote humoral immunity and are often associated with an immunosuppressive microenvironment (35, 36). Therefore, VISTA-induced Th2-biased differentiation via Tfr cells may further weaken the body’s anti-tumor immunity. This discovery provides a new explanation for understanding the mechanism of Th cell subset imbalance in ovarian cancer (18, 37, 38).

B cells participate in humoral immune responses through antibody secretion, and their functional abnormalities are closely associated with imbalances in the tumor microenvironment (28, 39). This study found that VISTA^+^ Tfr cells have no significant effect on B cell proliferation but specifically inhibit IgE secretion, with no significant regulatory effects on IgA, IgM, or IgG. This finding is unique, as previous studies have primarily focused on the general suppression of antibody secretion by Tfr cells, whereas this study is the first to identify selective regulation of IgE, which is dependent on VISTA (18, 40). The role of IgE in tumors is not fully understood, but it may participate in remodeling the immune microenvironment by activating mast cells to release cytokines (27). The inhibition of IgE by VISTA^+^ Tfr cells may indirectly affect inflammatory responses in the tumor microenvironment, though its specific pathological significance requires further investigation. Furthermore, this specific regulation suggests that the VISTA-Tfr cell axis may contribute to immune balance through fine-tuning of the antibody repertoire, providing a cellular-level perspective for understanding humoral immunitynd potential role in ovarian cancer.

At the molecular mechanism level, the regulation of different effector immune cells by VISTA^+^ Tfr cells exhibits significant “selectivity,” a phenomenon that may be related to differential expression of VISTA receptors on immune cell surfaces (41). Previous studies have confirmed that VISTA transmits inhibitory signals via receptors like PSGL-1 and VSIG3, whose expression varies across CD8^+^ T cells, CD4^+^CD25^−^ T cells, and B cells. This receptor heterogeneity may explain the selective regulatory effects of VISTA^+^ Tfr cells observed in our *in vitro* models (42). For example, CD8^+^ T cells highly express PSGL-1, while B cells express VSIG3 only at specific differentiation stages, which may explain why the regulation of B cells by VISTA^+^ Tfr cells is limited to IgE secretion (40, 43). Furthermore, the mode of interaction between Tfr cells and different effector cells (e.g., direct cell-to-cell contact or paracrine cytokine signaling) may also influence regulatory specificity: close contact with CD8^+^ T cells may facilitate efficient VISTA signal transmission through the “immune synapse” (44). In contrast, regulation of B cells may rely on soluble factors such as IL-10, and such differences could contribute to the selectivity of regulatory effects (39).

VISTA, as a novel B7 family checkpoint, has emerged as a promising target for cancer immunotherapy, with several VISTA inhibitors (monoclonal antibodies, small molecules, and fusion proteins) currently in preclinical or early clinical development (45). For example, anti-VISTA monoclonal antibodies have been shown to reduce intratumoral regulatory T cell infiltration and enhance IFN-γ secretion by CD8^+^ T cells (46, 47). Our findings that VISTA^+^ Tfr cells suppress effector immune cell function further support the rationale for VISTA-targeted therapies: inhibiting VISTA may not only directly activate T cells but also disrupt the immunosuppressive effects of Tfr cellstsuppress a “ells hitl to enhance anti-tumor immunity. Moreover, combination strategies (e.g., anti-VISTA + anti-PD-1) have shown synergistic effects in preclinical models, as VISTA and PD-1 may act through distinct signaling pathways to induce T cell exhaustion. Our observation that VISTA^+^ Tfr cells upregulate PD-1 on CD8^+^ T cells suggests that co-targeting VISTA and PD-1 could reverse Tfr cell-mediated immunosuppression more effectively than single-agent therapy. These findings highlight the potential of VISTA inhibitors as a complementary strategy to existing immunotherapies for ovarian cancer, a disease with limited response to PD-1/PD-L1 blockade alone.

Notably, the specific upregulation of exhaustion molecules (PD-1, CTLA-4) on CD8^+^ T cells by VISTA^+^ Tfr cells suggests that they may form a “synergistic network” with other immune checkpoints (27). Previous studies have shown that PD-1 and CTLA-4 exhibit cross-regulation in T cell exhaustion (48, 49). This study found that VISTA simultaneously upregulates both molecules, and silencing VISTA significantly reduced only PD-1 expression, suggesting that VISTA may be an upstream regulator of the PD-1 pathway (50). This finding provides experimental support for combination immunotherapy targeting both VISTA and PD-1/PD-L1apyE.DATA ot studies have confirmed that dual blockade can reverse T cell exhaustion and enhance anti-tumor responses (18, 51, 52). This study suggests that Tfr cells may be key target cells for this combination strategy.

Despite the insights gained, this study has several limitations. First, the research is conducted exclusively at the *in vitro* cellular level, using peripheral blood mononuclear cells (PBMCs) from healthy donors rather than ovarian cancer patients. This limits the direct translational relevance to the actual ovarian cancer microenvironment, where immune cells may exhibit distinct phenotypic and functional characteristics. Second, we did not validate the regulatory role of VISTA^+^ Tfr cells in *in vivo* ovarian cancer models (e.g., xenograft or syngeneic models), which is essential to confirm their contribution to tumor growth and immune escape in a physiological context. Third, the molecular mechanisms underlying the selective regulation of effector cells by VISTA^+^ Tfr cells (e.g., specific VISTA receptors on target cells) were not fully elucidated and require further investigation using receptor-blocking or knockout experiments. Future studies will address these limitations by incorporating patient-derived immune cells, *in vivo* models, and mechanistic validation to strengthen the clinical relevance of our findings.

## 5 Conclusion

In summary, this study reveals a novel mechanism by which VISTA^+^ Tfr cells contribute to immune escape in ovarian cancer through multi-dimensional regulation of effector immune cell functions, providing new perspectives for understanding the complexity of the tumor immune microenvironment. Future research could focus on the synergistic interactions between VISTA and other immune checkpoints, the influence of Tfr cell metabolic characteristics on their function, and the correlation between VISTA^+^ Tfr cells and patient outcomes in clinical samples. These explorations will promote the clinical translation of VISTA-targeted immunotherapy.

## Data Availability

The raw data supporting the conclusions of this article will be made available by the authors, without undue reservation.

## Supplementary material

Supplementary Figure 1 Image showing the markers after lentiviral transfection of Tfr cells.

Supplementary Figure 2 Regulatory Effects of VISTA^+^/VISTA^−^ Tfr Cells on CD8^+^ T Cell Immunity and Activation Status. **(A–D)** Levels of IL-2 **(A)**, IFN-γ **(B)**, perforin (PFP) **(C)**, and granzyme B **(D)** in culture supernatants of VISTA^−^ Tfr + CD8^+^ T cell group and VISTA^+^ Tfr + CD8^+^ T cell group as detected by ELISA (n=3). **(E–J)** Quantitative bar graphs showing the expression proportions of activation marker CD69 and exhaustion-associated immune checkpoints (LAG3, TIM-3, PD-1, CTLA-4) on CD8^+^ T cells in the co-culture system with VISTA^+^/VISTA^−^ Tfr cells, as detected by flow cytometry. The statistical results are expressed as the mean ± SD, **p* < 0.05, ***p* < 0.01.

Supplementary Figure 3 Regulatory Effects of VISTA^+^/VISTA^−^ Tfr Cells on Cytokine Secretion and Th Cell Differentiation of CD4^+^CD25^−^ T Cells. **(A–C)** Levels of IL-1α **(A)**, IL-2 **(B),** and TNF-α **(C)** in culture supernatants of VISTA^−^ Tfr + CD25^−^CD4^+^ T cell group and VISTA^+^ Tfr + CD25^−^CD4^+^ T cell group as detected by ELISA (n=3). **(D–F)** Quantitative bar graphs showing the expression proportions of differentiation markers IFN-γ, IL-4, and IL-17A on CD25^−^CD4^+^ T cells in the co-culture system with VISTA^+^/VISTA^−^ Tfr cells, as detected by flow cytometry. The statistical results are expressed as the mean ± SD, **p* < 0.05, ***p* < 0.01.

Supplementary Figure 4 Regulatory Effects of VISTA^+^/VISTA^−^ Tfr Cells on B Cell Proliferation and Antibody Secretion. **(A–D)** Levels of IgA **(A)**, IgE **(B)**, IgM **(C)**, and IgG **(D)** in culture supernatants of VISTA^−^ Tfr + B cell group and VISTA^+^ Tfr + B cell group as detected by ELISA (n=3). The statistical results are expressed as the mean ± SD, **p* < 0.05, ***p* < 0.01.
