# Supplementary material for: VISTA+ follicular regulatory T cells modulate the function of effector immune cells: implications for ovarian cancer immune escape
Source: Front Immunol. 2025 Nov 28;16:1704048. doi: 10.3389/fimmu.2025.1704048 (PMC12698577; doi:10.3389/fimmu.2025.1704048)

Supplementary Figure 1

CD8⁺T cell

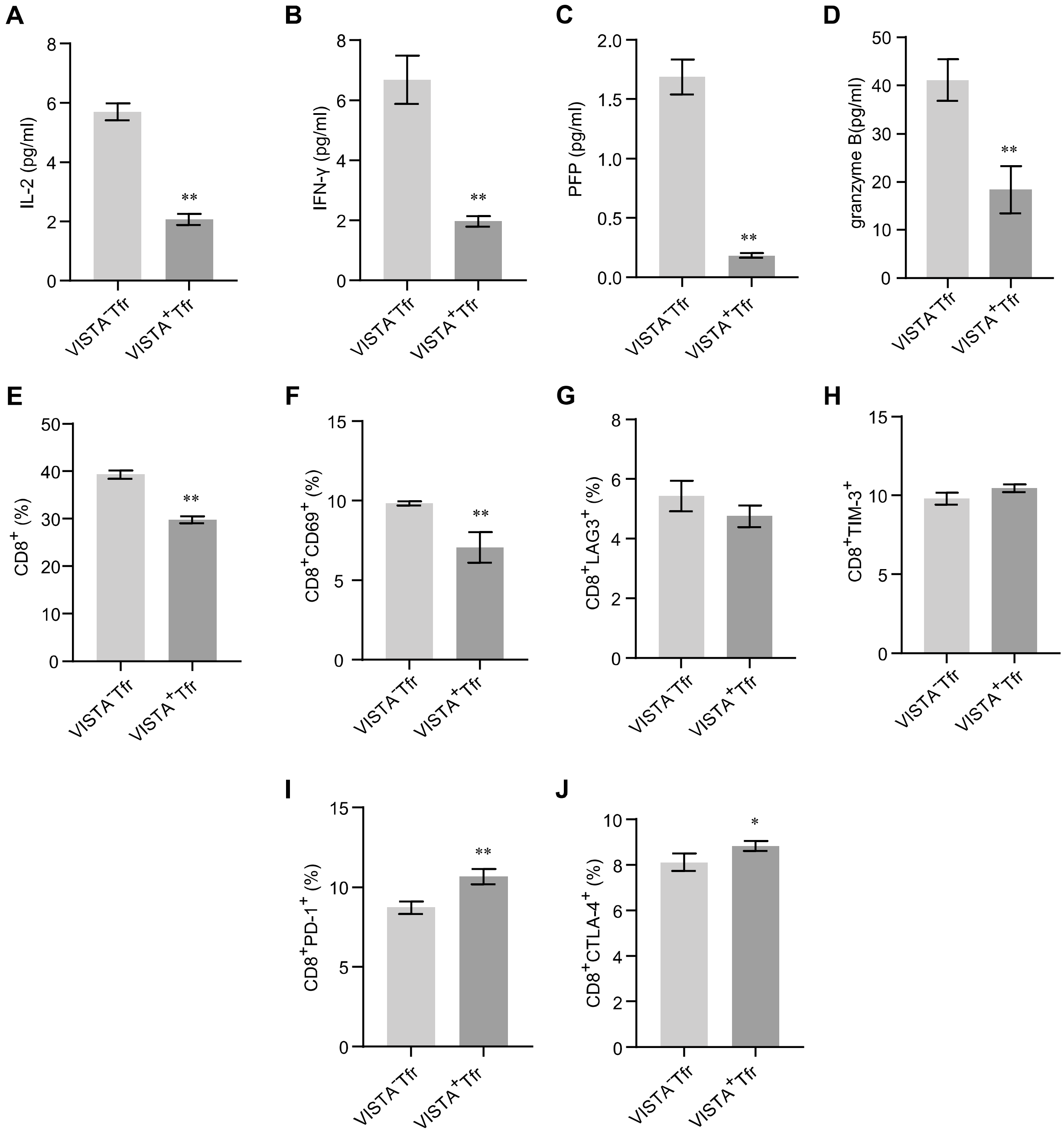

Supplementary Figure 2

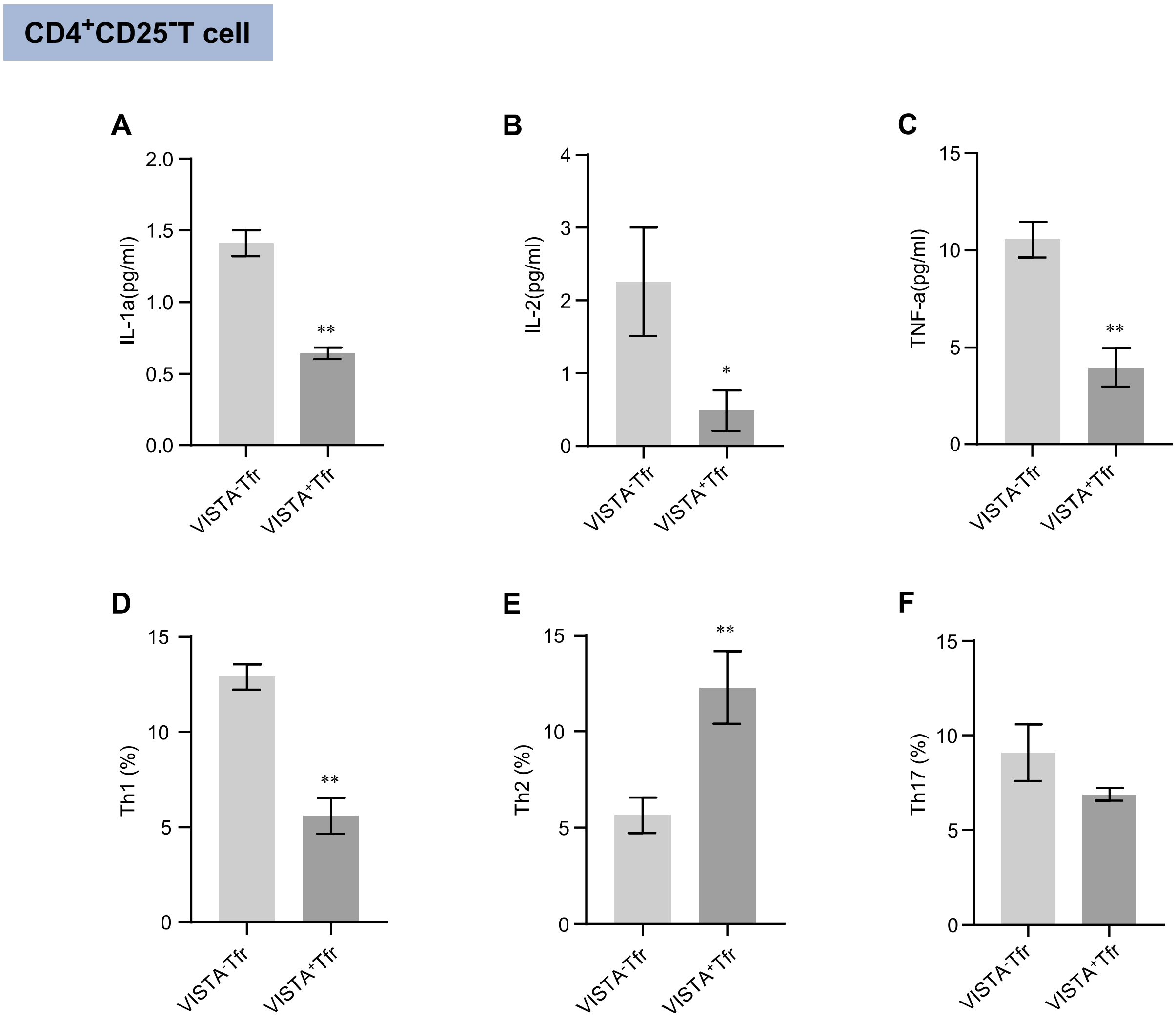

Supplementary Figure 3

B cell

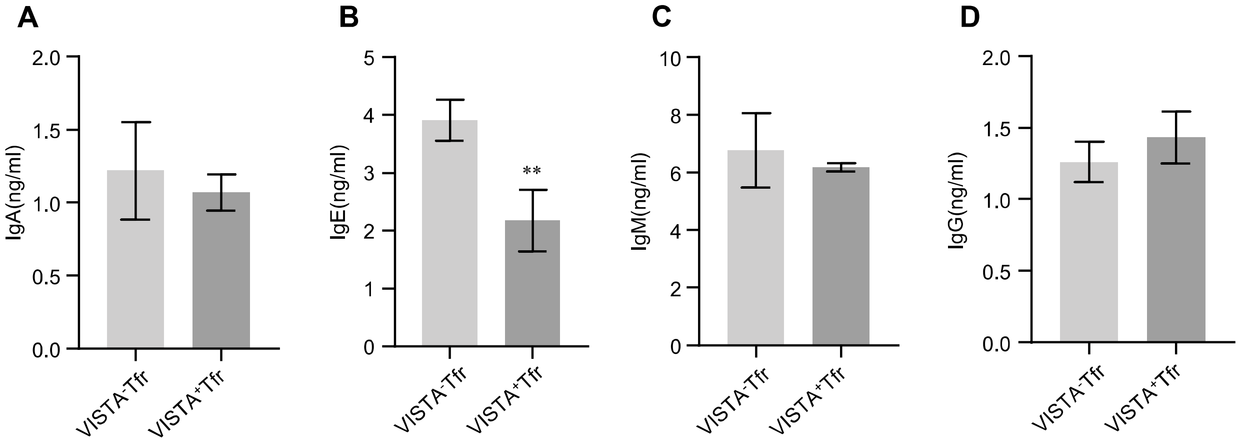

Supplement: Supplementary Figure 1 — Image showing the markers after lentiviral transfection of Tfr cells. [file Image1.pdf]
